# Molecular determinants of mechanosensation in the muscle spindle

**DOI:** 10.1016/j.conb.2022.102542

**Published:** 2022-04-14

**Authors:** Katherine A. Wilkinson

**Affiliations:** Department of Biological Sciences, San José State University, San Jose, CA, USA

## Abstract

The muscle spindle (MS) provides essential sensory information for motor control and proprioception. The Group Ia and II MS afferents are low threshold slowly-adapting mechanoreceptors and report both static muscle length and dynamic muscle movement information. The exact molecular mechanism by which MS afferents transduce muscle movement into action potentials is incompletely understood. This short review will discuss recent evidence suggesting that PIEZO2 is an essential mechanically sensitive ion channel in MS afferents and that vesicle-released glutamate contributes to maintaining afferent excitability during the static phase of stretch. Other mechanically gated ion channels, voltage-gated sodium channels, other ion channels, regulatory proteins, and interactions with the intrafusal fibers are also important for MS afferent mechanosensation. Future studies are needed to fully understand mechanosensation in the MS and whether different complements of molecular mediators contribute to the different response properties of Group Ia and II afferents.

## Introduction

The muscle spindle (MS) is an encapsulated sensory organ located in parallel to the extrafusal muscle fibers. It is composed of contractile intrafusal bag and chain fibers whose polar regions are innervated by static and dynamic gamma motor neurons that control the intrafusal fiber length and by this maintain their sensitivity in all contractile states [1]. The MS is also innervated by stretch-sensitive Group Ia and II afferents. These slowly adapting low threshold mechanoreceptors report static muscle length as well as muscle movement. The Group Ia and II MS afferents differ in their dynamic and static sensitivities to stretch, innervation patterns, and gene expression patterns [1-3••]. The MS afferents are the sensory part of the myotatic reflex, which is important for motor control, coordinated movements, and balance. MS afferent sensory information also provides the primary input for proprioception, the sense of body and limb position in space [4]. The molecular mechanisms by which MS afferents transduce muscle movement into action potentials are incompletely understood, although a variety of ion channels, synaptic-like vesicles, and interactions with the intrafusal fibers are thought to be necessary [5]. Here I will discuss the recent evidence for molecular elements thought to be involved and identify some unanswered questions in MS mechanosensation.

## PIEZO2 is necessary for MS afferent mechanosensation

The PIEZO2 channel is a rapidly adapting, mechanically sensitive, non-selective cation channel that has been implicated in mechanosensation in a wide range of cell types [6], including muscle proprioceptors [7,8] and the Merkel cell–neurite complex, which is also a slowly adapting low threshold mechanoreceptor [9,10]. Absence of PIEZO2 in dorsal root ganglion (DRG) or mesencephalic trigeminal nucleus proprioceptors in mice eliminates the rapidly adapting mechanically sensitive current in cell bodies and causes defects in balance and limb coordination [7,8]. MS anatomy and innervation appear normal, but almost no stretch-sensitive activity can be recorded from MS afferents lacking *PIEZO2*, suggesting the need for PIEZO2 in afferent endings for proper function [7]. Loss of *PIEZO2* in proprioceptors also leads to scoliosis and hip dysplasia, similar to what was seen in mice completely lacking proprioceptive innervation [11•]. The human PIEZO2 protein is highly homologous to mouse PIEZO2 [12] and mutations causing loss of function in PIEZO2 have been associated with rare genetic diseases causing proprioceptive deficits (reviewed in Ref. [6]). The similarity in symptoms seen in human patients and mouse models following loss of the *P1EZO2* gene argue that PIEZO2 is also necessary for normal MS afferent function in humans.

PIEZO2’s rapidly inactivating currents in proprioceptor somas do not match the slowly adapting currents elicited in response to stretch, though. It is unclear how similar soma PIEZO2 channel kinetics are to those of the channel in the afferent endings, but the differences might suggest the presence of modulatory influences. One modulator is the molecular environment in which the PIEZO2 channel is embedded. Unlike PIEZO1 which can be opened by force-from-lipids and lipid membrane stretch alone [13], PIEZO2 activity seems to rely both on the mechanics of the plasma membrane as well as force-from-filament forces from the cytoskeleton and extracellular matrix [6,14,15]. PIEZO2 can be activated by membrane indentation on proprioceptor cell bodies [7] as well as increased static plasma tension caused by osmotic swelling [16]. Membrane composition and lipid bilayer rigidity also alter PIEZO2 mechanosensitivity with margaric acid and decreased levels of phosphoinositides inhibiting and stomatin-like protein 3 (*Stoml3*) and cholesterol-rich lipid rafts increasing PIEZO2 currents [14,17-19]. An intact cytoskeleton is also necessary for PIEZO2 mechanotransduction, as disrupting actin or microtubule polymerization decreases PIEZO2 currents [14,16,20,21]. Conversely, PIEZO2 currents in Merkel cells were potentiated in the presence of paclitaxel, which prevents microtubule depolymerization [21].

Additional proteins in the afferent endings may also modulate PIEZO2 channel kinetics and contribute to the slowly adapting response in MS afferents. TMEM150c/Tentonin-3 was thought to be a mechanically sensitive ion channel, but mechanosensitive currents in TMEM150C expressing HEK cells are eliminated following the elimination of endogenous PIEZO1 expression [22], suggesting that TMEM150C is not a channel but modulates mechanotransduction by PIEZO channels. In agreement with this idea, TMEM150c is not preferentially expressed in neurons with mechanosensitive currents and selective knockdown of TMEM150C using siRNA does not alter mechanosensitivity in DRG neuron soma [23]. However, others have shown slowly adapting mechanical current in Piezo1 deficient cells treated with actin-stabilizing agents [24], so whether TMEM150c can act as an independent mechanically sensitive ion channel in proprioceptors is still unresolved. TMEM150c is present in MS afferent endings and loss of TMEM150c leads to proprioceptive deficits and lower MS afferent firing rates during stretch [25]. These effects could be due to the ability of TMEM150C to enhance PIEZO2 currents both by prolonging the time to inactivation and decreasing the activation threshold [26•]. Future studies are needed to resolve whether TMEM150C and/or additional proteins in afferent endings modulate PIEZO2 mechanotransduction and allow for additional mechanosensitive current during prolonged stretch.

## Role of additional mechanically sensitive ion channels

Additional mechanically sensitive ion channels, including members of the DEG/ENaC and TRP families, have been identified in the MS afferents by immunohistochemistry and/or RNA sequencing [2••,^27^], but their role in mechanosensation is even less well understood. The spinal curvature phenotype in mice lacking *PIEZO2* in proprioceptors was slightly different from that seen in *Runx3* knockout mice, which lack all proprioceptive innervation [11•], suggesting the presence of additional mechanically sensitive ion channels that could compensate for some of the effects of gene ablation. Additional mechanically sensitive ion channels are ideally situated to contribute ionic current to maintain firing during sustained stretch. As potassium current does not seem to underlie the abrupt cessation of firing upon the release of stretch [28], it seems likely that the closing of mechanically sensitive sodium or cation channels is necessary to mediate the abrupt cessation of firing when the muscle is shortened. If PIEZO2 is as rapidly adapting in the afferent endings as it is in the soma, additional mechanically sensitive ion channels would have to mediate the cessation of firing after stretch. Using RNAseq, six mechanically sensitive ion channels were shown to be differentially expressed in MS afferent subtypes. Eight other mechanically sensitive ion channels, including PIEZO2, were found in all proprioceptor subtypes [2••]. This could suggest that different complements of mechanically sensitive ion channels help differentiate mechanosensitivity in MS afferent subtypes, but this possibility remains to be experimentally tested. Loss of the scaffolding protein Whirlin decreases stretch evoked firing and firing fidelity [29]. Whirlin is known to interact with mechanically sensitive ion channels in other cell types [30] but its interactome in MS afferent endings or whether it has a role in regulating the subcellular distribution of the channels within the endings remain to be determined [29].

Members of the DEG/ENaC family are the candidate mechanosensitive ion channels with the most convincing evidence for contributing to MS afferent mechanosensation. The α, β, and γ subunits of the ENaC channels and ASIC2 and ASIC3 have been localized to afferent endings using immunohistochemistry [27,31]. ASIC1, ASIC2, and ASIC3 have also been shown to be expressed in MS afferents using RNA sequencing [2••]. Functionally, knockout of ASIC3 in parvalbumin-expressing proprioceptors decreases substrate-driven neurite stretch response in cultured DRG neurons and causes proprioceptive impairments *in vivo*, especially in the dark. Interestingly, though, MS afferent firing was increased during dynamic stretch but otherwise unchanged [31]. These results could have been due to compensation by other ASIC subunits or because ASIC3 is found primarily in Group II MS afferents so the population responses are skewed towards more dynamically sensitive Group Ia MS afferents in the knockout animals [2••,^31^]. Amiloride, a non-specific blocker of DEG/ENaC channels, and its analogs can decrease MS spindle afferent firing, but caution should be taken when interpreting these results as DEG/ENaC channels are also found in both intrafusal and extrafusal muscle fibers so the drug effects could have been due to changes in muscle tone [27]. The MS afferent receptor potential is primarily sodium, so the DEG/ENaC channels are well suited to allow for more sodium current in addition to the mixed cation current from PIEZO2 [28]. Additional functional studies are needed to clarify the role of the DEG/ENaC channels in MS mechanosensation.

## Vesicle-released glutamate is necessary for maintaining MS afferent excitability

**Figure 1:**
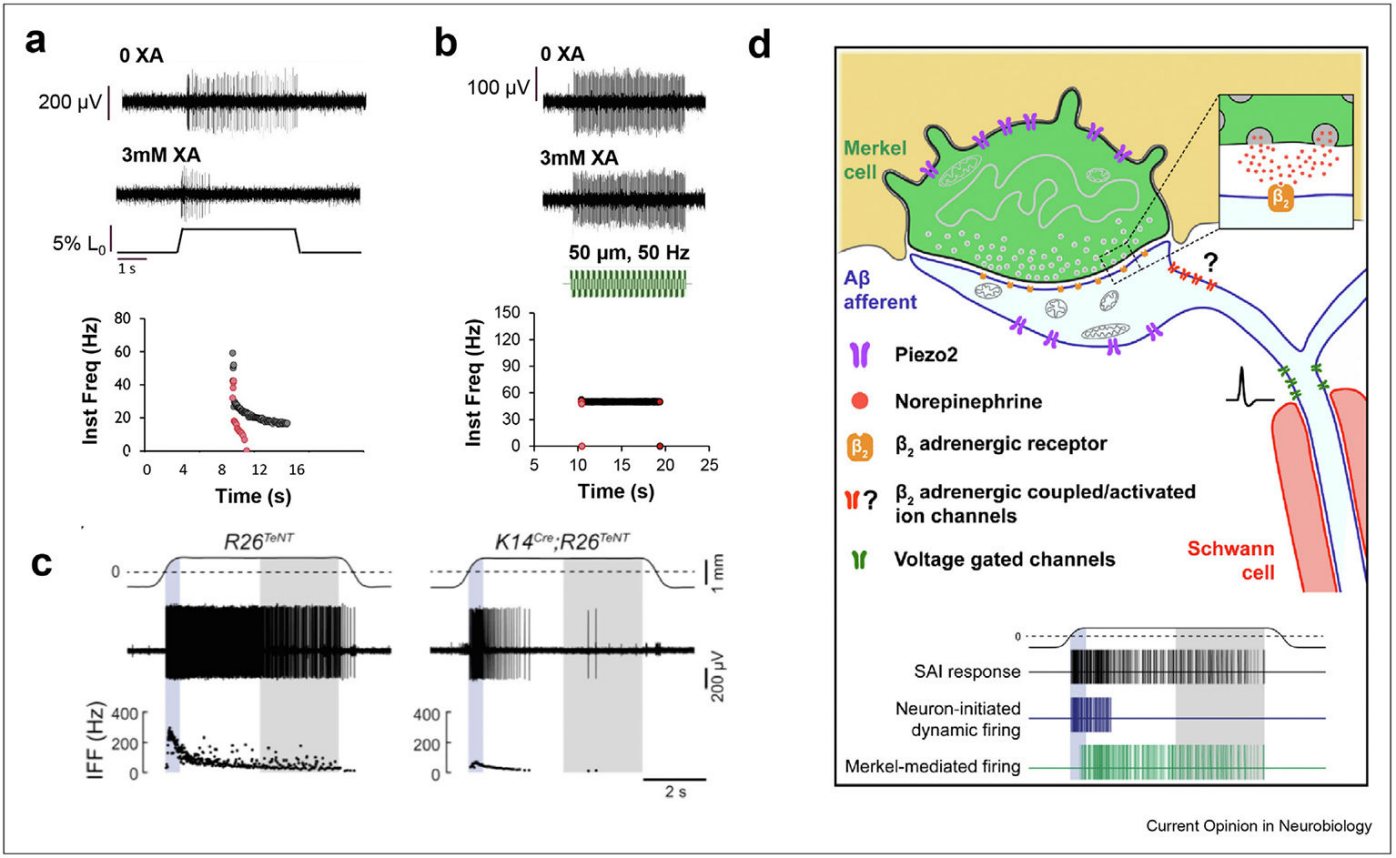
Similarity in mechanosensation in the muscle spindle and Merkel cell-neurite complex. Xanthurenic acid (XA) was used to block the packaging of glutamate into synaptic-like vesicles and MS afferent response to ramp-and-hold stretch and sinusoidal vibration assayed before and after XA. MS afferent firing was decreased or eliminated during ramp-and-hold stretch in the majority of afferents tested and firing at the end of stretch was affected earliest (**a**). Even in some units that could not maintain firing during stretch, the response to vibration was unchanged (**b;** same unit as **a;** [34•]), suggesting vesicle-released glutamate is required for static but not dynamic response to stretch likely via effects on general afferent excitability, (**c**) Similarly, in the Merkel cell-associated Aβ afferent, preventing the Merkel cell from releasing synaptic vesicles (K14^Cre^;R26^TeNT^) preferentially decreased firing during the hold phase of touch as compared to littermate controls (R26^TeNT^). A similar reduction in static touch response occurs if Merkel cells are eliminated [37], *PIEZO2* in Merkel cells is eliminated [9,10], or the β2 receptor is eliminated from the Aβ afferent [39], (**d**). The mechanosensation model proposed for the Merkel cell–neurite complex [39] is similar to that proposed here for the MS afferent. Touch is thought to open PIEZO2 channels in the Aβ afferent to mediate the initial response. Opening of PIEZO2 in the Merkel cell then leads to synaptic-like vesicle release which is necessary for the static phase response via some unknown pathway. Panels **a** and **b** taken from [34•] and **c** and **d** from Ref. [39].

MS afferent endings contain glutamate-filled synaptic-like vesicles that are released in a stretch and calcium-dependent manner [32]. The primary transporter transferring glutamate from the cytoplasm into the vesicles in MS afferent endings is the vesicular glutamate transporter 1 (VGLUT1) [33]. Blocking VGLUT1 using xanthurenic acid leads to decreased afferent excitability. Decreased firing was observed particularly during the static phase of a ramp-and-hold stretch, whereas firing during the dynamic phase of the stretch and during sinusoidal vibration were less affected (Figure 1a-b). Similarly, animals lacking one copy of the *VGLUT1* gene had lower firing rates during the plateau phase of ramp-and-hold stretches but similar firing rates during vibration as wild-type controls, confirming that glutamate release is essential for static but not dynamic sensitivity [34•]. As calcium has been shown to enter the sensory terminal upon stretch, this vesicle-released glutamate is ideally situated to couple mechanically generated receptor potentials with additional depolarizing current to maintain firing during sustained stretch. Calcium ions could enter directly through PIEZO2 but other voltage-gated calcium channels might also contribute.

The time-course of vesicle release is such that glutamate is likely to primarily act by increasing overall afferent excitability, with the kinetics of afferent firing determined primarily by the mechanically sensitive ion channels and potentially voltage-gated channels. Vesicle-released glutamate presumably acts on an autoreceptor on the afferent ending, but the identity of the glutamate receptor(s) is/are still to be determined. Ionotropic glutamate receptor(s) could allow for the quick flow of additional sodium current, while metabotropic receptors would suggest modulatory effects on other ion channels. Antagonists to the phospholipase-D coupled metabotropic glutamate receptor (PLD mGluR) could block all stretch-sensitive firing, but it took almost 4 h to do so, which is much longer than the minutes required for effects to be seen after blocking glutamate packaging [34•]. Kainic acid was also reported to increase the firing rate in some [32,35], but not all studies [34•], with the discrepancy potentially due to receptor desensitization. All other drugs tested targeting ionotropic and metabotropic glutamate receptors were ineffective, however, only whole nerve firing in response to stretch and not the firing rates of identified MS afferents were measured. Additionally, these tests had a relatively low sample size (4–6), so subtle changes in MS afferent firing could have been missed [32]. Recent single-cell RNAseq studies in proprioceptors have identified glutamate receptor coding genes including NMDA, AMPA, and kainate receptor subunits, mGluR4, mGluR7, and mGluR8 [2••,3••]. These results are likely biased towards receptor subtypes expressed in the soma, may miss genes present at low frequencies, and do not provide information about the expression at the afferent ending. Immunohistochemistry on rat masseter muscle MS afferent endings identified mGluR5 expression, but not NMDAR2B or GluR1 [36]. Once the glutamate receptor(s) involved are identified, the specific role of vesicle-released glutamate in MS afferent mechanotransduction will be better understood.

Interestingly, a similar role for vesicle-released neurotransmitters has been identified in the Merkel cell–neurite complex. Elimination of Merkel cells [37] or of PIEZO2 expression in Merkel cells [9,10] leads to firing only at the beginning phase of the touch response. This firing is due to the mechanically sensitive Aβ low threshold mechanoreceptors (LTMRs), which also express PIEZO2 [38]. Merkel cells also contain the machinery to release synaptic-like vesicles, and blocking vesicle release phenocopied the effect of eliminating Merkel cells entirely (Figure 1c) [39]. This suggests that Merkel cell release of neurotransmitters is also important for static phase firing of LTMRs. However, the identity of the neurotransmitter(s) involved and how these effects are mediated is still controversial. One study suggests that norepinephrine acting via the β_2_ receptor is necessary to mediate prolonged static phase firing in the Merkel-cell afferents ([39]; Figure 1d). However, other studies have implicated Merkel cell glutamate or serotonin release, and not norepinephrine, as necessary for full touch sensitivity [40,41]. In conclusion, both MS afferents and the Merkel-cell neurite complex appear to use similar strategies of vesicularly releasing modulatory components to generate slowly adapting responses while relying on a rapidly adapting mechanically sensitive ion channel (Figure 1). The mechanisms by which these modulatory components can help to mediate the unique slowly adapting firing properties of MS afferents and Merkel-cell neurite complexes remain to be determined.

## Contributions of voltage-gated ion channels to MS afferent mechanosensation

The complement of voltage-gated and other ion channels on the afferent endings, heminodes, and preterminal axons contributes to the overall excitability and neuronal firing properties of MS afferents (reviewed in more detail in Ref. [5]). Persistent inward sodium currents (INaP) have been implicated as important for sustained firing in MS afferents as tonic firing can be blocked using riluzole and phenytoin, which share only the ability to block INaP [42]. Na_v1.6_ and Na_v1.7_ immunoreactivity at heminodes and preterminal axons is observed, suggesting a role for those channels in spike initiation and maintenance of repetitive firing. More unexpectedly, Na_v1.1_, Na_v1.6_, and Na_v1.7_ are observed in the sensory encoding regions of the MS afferent endings, suggesting that INaP from these channels could amplify the receptor current as it travels to the heminodes [43••]. More experiments are needed to clarify the role of each of the Na_v_ channels in the generation of the receptor current, spike initiation, and tonic firing in MS afferents.

Blocking calcium and calcium-activated potassium channels, K(Ca), can alter MS afferent dynamic sensitivity, although the specific channels affected have not yet been identified [44]. The K(Ca) channel SK2 has been observed in MS afferent endings, but functional studies are necessary to understand its role in mechanosensation [45]. Multiple voltage-gated sodium, potassium, and calcium channels are found using RNA sequencing in proprioceptors, although it remains to be determined which channels are localized to afferent endings [2••,3••,^46^]. Interestingly, though, MS afferent subtypes show differential expression of K_v_ channels, notably with Group Ia afferents preferentially expressing Kv_1.1_ and Kv_1.2_ [2••,3••]]. Inhibiting the Kv_1.1_ and Kv_1.2_ channels in DRG soma changes phasic firing in response to current injection from putative Group Ia afferents to tonic firing [3••]. Whether this differential expression of K_v_ channels occurs on the soma and/or afferent endings and contributes to the increased dynamic sensitivity seen in Group Ia afferents as compared to Group II afferents is currently unknown [3••].

## Intrafusal fiber and MS afferent interactions are important for mechanosensation

The MS is composed of chain fibers and 2 types of bag intrafusal fibers which are differentially innervated by static or dynamic gamma motor neurons. The fiber types that an MS afferent innervates contribute to their dynamic and static sensitivities, although the relative contribution of intrafusal fibers compared to afferent mechanisms is not well understood [1,44]. Recent evidence suggests that in addition to the role of intrafusal fiber mechanics on afferents, there is also cholinergic signaling to the intrafusal fibers from the MS afferents [47••,^48^]. MS afferents contain the machinery necessary to release synaptic-like vesicles containing acetylcholine and the contact site on the intrafusal fibers in the equatorial region of the spindle contain acetylcholine receptors [48]. Blocking acetylcholine receptors or packaging and release of acetylcholine in an *ex vivo* muscle nerve preparation with no gamma motor tone increased MS afferent firing, presumably by altering intrafusal fiber tone, although this remains to be experimentally shown [47••]. Future work is needed to understand the role of this acetylcholine signaling pathway during normal movement and disease.

Intrafusal fibers contain many of the same proteins as extrafusal fibers [49], but until recently, MS effects during neuromuscular diseases have been largely overlooked. In two mouse lines with mutations modeling different forms of muscular dystrophy, increases in MS afferent response at rest and during sinusoidal vibration were observed even though intrafusal fibers structurally seemed less affected than extrafusal fibers [50]. Similarly, in a mouse model of Amish Nemaline Myopathy, loss of the slow skeletal muscle isoform of troponin T caused changes in intrafusal nuclear bag fibers and deficits in performance on the rotarod and balance beam [51]. Additional neuromuscular diseases likely also have MS impairments and understanding those effects may suggest therapeutic treatments as well as provide insight into the regulation of mechanosensation by intrafusal fibers [52].

## Summary

**Figure 2:**
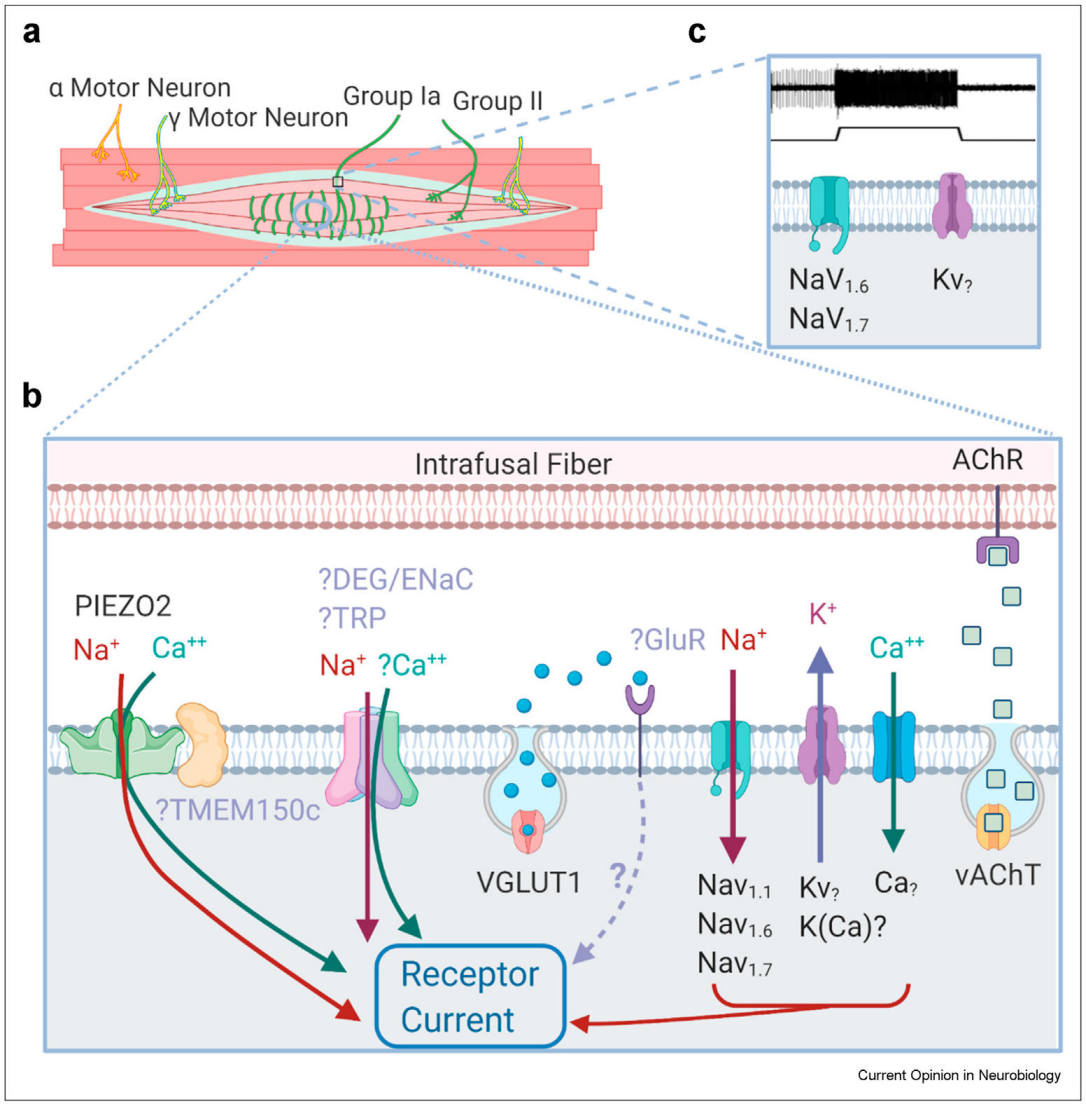
Molecular contributors to mechanosensation in muscle spindle afferents. (**a**) Schematic of the muscle spindle (MS), which is innervated by Group Ia and II MS afferents as well gamma motor neuron efferents. (**b**) Sensory endings in the MS afferent (gray) require the mechanically sensitive non-specific cation channel PIEZO2 for normal function [7]. TMEM150c/Tentonin-3 is found in MS afferent endings and has been shown to enhance PIEZO2 current and increase the time to inactivation [25,26•]. Additional mechanically sensitive ion channels have been found in MS afferents, including DEG/ENaC and TRP family members, but future work is needed to understand their role in mechanosensation [2••,^27^,^31^]. Synaptic-like vesicles containing glutamate are released in a stretch and calcium-dependent manner and are necessary for maintaining afferent excitability and static sensitivity [32,34•]. The glutamate receptor(s) (GluR) and signaling pathway(s) necessary to mediate the glutamate-induced effects are currently unknown. Voltage-gated sodium channels (Nav) are located on MS afferent sensory endings and presumably amplify receptor current as it travels to the spike generating heminode [43••]. Additional ion channels are necessary for receptor current generation and different complements of ion channels may underlie differences in sensitivity of MS afferent subtypes [3••]. Mechanical interactions with the intrafusal fiber bag and chain fibers are also important for MS afferent mechanosensation. Acetylcholine is released from the MS afferent ending and binds to acetylcholine receptors on intrafusal fibers and decreases afferent sensitivity [47••]. (**c**) The heminode is the site of action potential generation and the complement of Nav and potassium channels and other ion channels can shape the slowly adapting response of the MS afferent to stretch ([5,43••]; raw trace of MS afferent response to stretch in mouse shown above). Abbreviations: VGLUT1 (vesicular glutamate transporter 1), VAChT (vesicular acetylcholine transporter), GluR (glutamate receptor), AChR (acetylcholine receptor). Figure modified from [34•]. Created with BioRender.com.

Recent work has suggested some key players in MS afferent mechanosensation (Figure 2), but there are still many unanswered questions about the identities and exact role of the proteins involved. Current evidence suggests that the primary mechanically sensitive ion channel in MS afferent endings is PIEZO2 [7,8], but that, additional molecular mediators are necessary given that the MS afferent receptor potential is primarily sodium [28]. Additional mechanically sensitive ion channels from the DEG/ENaC and TRP families may also be necessary for normal MS afferent mechanosensation [2••,^27^,^31^], potentially by providing additional sodium current and/or by modulating the dynamic sensitivity of the afferent. Additional molecular mediators including vesicle-released glutamate [34•], Na_v_ ion channels [43••], TMEM150c [25], Whirlin [29], and others are necessary for the generation of the slowly adapting response of MS afferents. The complement of sodium, potassium, and calcium ion channels at the sensory endings, heminodes, and axons are also important and differences in these channels may contribute to the different responses of Group Ia and Group II afferents [2••,3••,^5^,43••]]. Interactions with the intrafusal fibers, including a recently discovered acetylcholine signaling pathway [47••], also regulate MS afferent sensitivity. Variations in the complement of molecular mediators found on Group Ia vs. Group II afferents may help explain their unique response properties to stretch [2••,3••,^53^]. Similarly, changes in gene expression over time as well as structural changes may underlie the developmental differences in afferent responsiveness [2••,3••,^53^]. Other contributing ion channels and signaling pathways are waiting to be discovered to provide a more complete picture of how the complex MS afferent responses to muscle movement are generated.
